# Patterns of better breast cancer care in countries with higher human development index and healthcare expenditure: Insights from GLOBOCAN 2020

**DOI:** 10.3389/fpubh.2023.1137286

**Published:** 2023-04-12

**Authors:** Sina Azadnajafabad, Sahar Saeedi Moghaddam, Esmaeil Mohammadi, Sina Delazar, Sina Rashedi, Hamid Reza Baradaran, Morteza Mansourian

**Affiliations:** ^1^Department of Epidemiology, School of Public Health, Iran University of Medical Sciences, Tehran, Iran; ^2^Non-communicable Diseases Research Center, Endocrinology and Metabolism Population Sciences Institute, Tehran University of Medical Sciences, Tehran, Iran; ^3^Kiel Institute for the World Economy, Kiel, Germany; ^4^Department of Neurosurgery, University of Oklahoma Health Sciences Center (OUHSC), Oklahoma, OK, United States; ^5^Advanced Diagnostic and Interventional Radiology Research Center (ADIR), Imam Khomeini Hospital, Tehran University of Medical Sciences, Tehran, Iran; ^6^Rajai Cardiovascular Medical and Research Center, Iran University of Medical Sciences, Tehran, Iran; ^7^Ageing Clinical and Experimental Research Team, Institute of Applied Health Sciences, University of Aberdeen, Aberdeen, United Kingdom; ^8^Health Promotion Research Center, Iran University of Medical Sciences, Tehran, Iran

**Keywords:** breast cancer, epidemiology, mortality, incidence, quality of care, health economics, HDI

## Abstract

**Background:**

The huge burden of breast cancer (BC) necessitates the profound and accurate knowledge of the most recent cancer epidemiology and quality of care provided. We aimed to evaluate BC epidemiology and quality of care and examine the effects of socioeconomic development and healthcare expenditure on disparities in BC care.

**Methods:**

The results from the GLOBOCAN 2020 study were utilized to extract data on female BC, including incidence and mortality numbers, crude rates, and age-standardized rates [age-standardized incidence rates (ASIRs) and age-standardized mortality rates (ASMRs)]. The mortality-to-incidence ratio (MIR) was calculated for different locations and socioeconomic stratifications to examine disparities in BC care, with higher values reflecting poor quality of care and vice versa. In both descriptive and analytic approaches, the human development index (HDI) and the proportion of current healthcare expenditure (CHE) to gross domestic product (CHE/GDP%) were used to evaluate the values of MIR.

**Results:**

Globally, 2,261,419 (95% uncertainty interval (UI): 2,244,260–2,278,710) new cases of female BC were diagnosed in 2020, with a crude rate of 58.5/100,000 population, and caused 684,996 (675,493–694,633) deaths, with a crude rate of 17.7. The WHO region with the highest BC ASIR (69.7) was Europe, and the WHO region with the highest ASMR (19.1) was Africa. The very high HDI category had the highest BC ASIR (75.6), and low HDI areas had the highest ASMR (20.1). The overall calculated value of female BC MIR in 2020 was 0.30, with Africa having the highest value (0.48) and the low HDI category (0.53). A strong statistically significant inverse correlation was observed between the MIR and HDI values for countries/territories (Pearson's coefficient = −0.850, *p*-value < 0.001). A significant moderate inverse correlation was observed between the MIR and CHE/GDP values (Pearson's coefficient = −0.431, *p*-value < 0.001).

**Conclusions:**

This study highlighted that MIR of BC was higher in less developed areas and less wealthy countries. MIR as an indicator of the quality of care showed that locations with higher healthcare expenditure had better BC care. More focused interventions in developing regions and in those with limited resources are needed to alleviate the burden of BC and resolve disparities in BC care.

## Introduction

Breast cancer (BC) ranks as the leading cause of malignancy in women and has placed a massive burden on the healthcare systems (1, 2). According to the latest Global Cancer Statistics (GLOBOCAN) 2020 estimates, female BC surpassed lung cancer in terms of the cancer type with the highest incidence globally, with ~2.3 million new cases diagnosed in 2020 (1). Over the past three decades, the number of new cases and age-standardized rates for BC have been on increasing trends, indicating an alarming pattern (2, 3). In comparison, although the global number of deaths and disability-adjusted life years (DALYs) due to BC increased, the age-standardized values for these measures decreased slightly (2, 3). There are significant geographic and socioeconomic variations in the incidence and burden of BC on global, regional, and national scales (3–5).

The quality of cancer care provided to patients with cancers directly affects individual outcomes of treatment (6) and, on a larger dimension, contributes to population trends in cancer outcomes (7). In this regard, global cancer quality assessment and measurement programs/tools have been developed to measure and improve the care provided to patients (8, 9). This evaluation was done by examining epidemiological data and population health metrics and *via* proxies that may reflect the overall quality of care provided (10, 11). One of the well-known and validated proxies of the quality of care, especially for cancer causes, is the mortality-to-incidence ratio (MIR), which has been shown to be a beneficial indicator of global cancer screening and care (11). To date, MIR has been recruited on various aggregated databases to examine the cancer care status and disparities among different geographic and socioeconomic categories for several cancers, including colorectal (11, 12), liver (13), pancreatic (14), gastric (15, 16), breast (17, 18), prostate (19), and kidney cancers (20, 21), and the results have been encouraging.

Both the epidemiology and quality of BC care are affected by the socioeconomic status, the degree of development of countries, and the ability of the corresponding health system to be responsible for providing health coverage and services (2, 3). The literature suggests that countries and regions with a better socioeconomic ranking have better care for BC, better control of risk factors that contribute to disease development, and *vice versa* (2). The human development index (HDI) is one of the most successful measures of socioeconomic development deployed to compare health disparities in recent years (22, 23). In addition, countries with high healthcare expenditure and government investments in healthcare have shown favorable cancer outcomes for various malignancies (12, 16, 20, 24).

To address the abovementioned factors, health inequalities and disparities among various populations with diverse socioeconomic states have been studied. In the case of BC, it was suggested that socioeconomic factors may influence cancer biology and lead to health disparities (25, 26). In the globalization period that the world is experiencing, global health inequalities are significant in developing countries where the healthcare systems are not competent enough to cope with the burden of disease and cancer (27). Regarding the indicators chosen to investigate disparities in BC care in this study, the role of economic factors in access to BC screening, diagnosis, and treatment has been proven in terms of reflection on the number of physicians and imaging facilities (28–30). Therefore, it is essential to provide decent evidence on existing inequalities and disparities in BC care to help policymakers cover the gaps by distributing clinical expertise and health infrastructure more equally around the world (31).

In this study, we aimed to present the most recent female BC epidemiology and corresponding MIR using the results from the GLOBOCAN 2020 study and the associations of MIR of BC with HDI and healthcare expenditure to examine the impact of these factors on global BC care and to map disparities in BC care for the benefit of healthcare policymakers and regional and national authorities.

## Methods

### Data source

**Figure 1:**
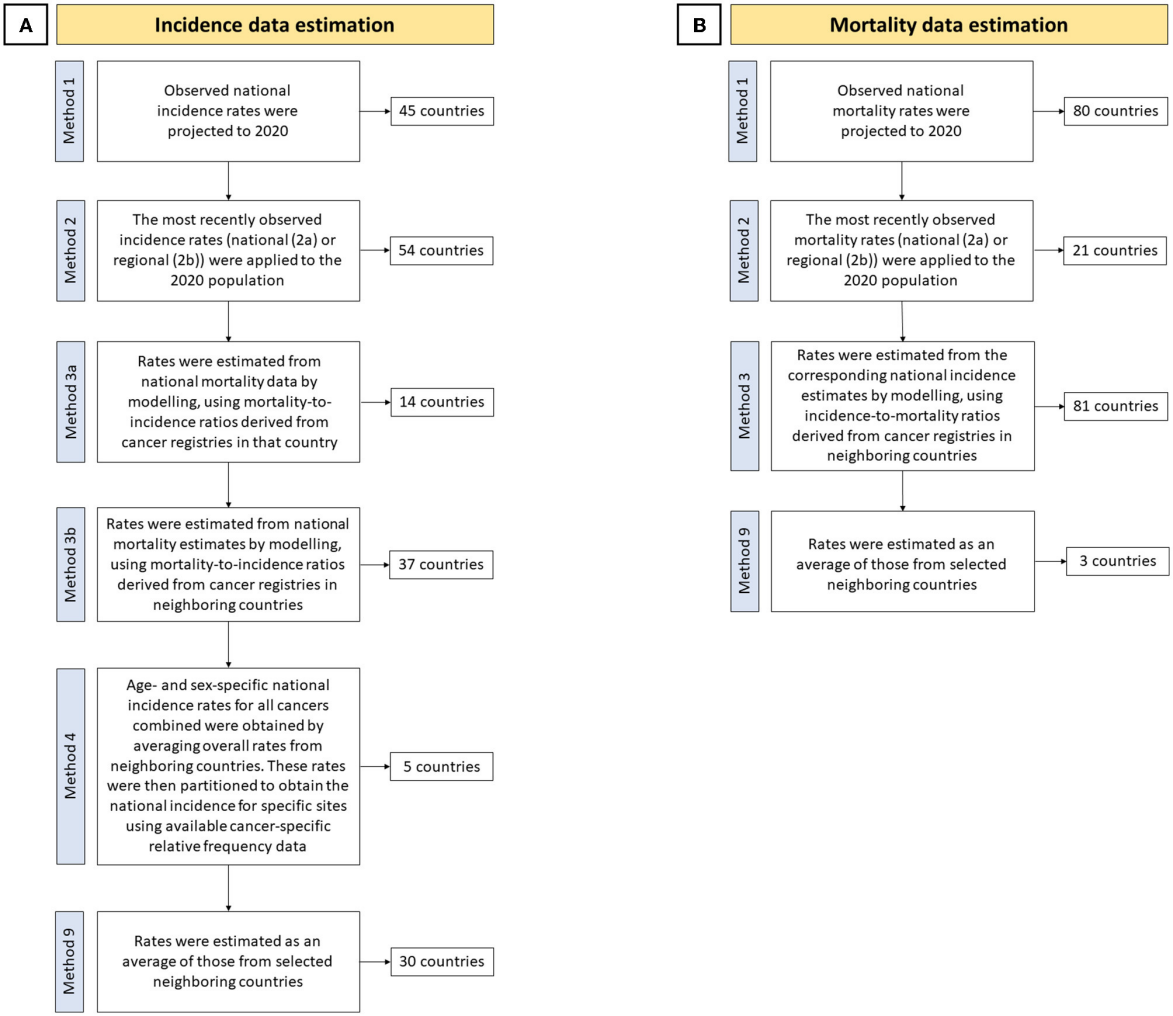
Cancer **(A)** incidence and **(B)** mortality estimation steps and methods in the GLOBOCAN 2020 study (34).

The results from the GLOBOCAN 2020 study on cancer epidemiology estimates were reported in this study (available at: https://gco.iarc.fr/today). GLOBOCAN is an endeavor of the International Agency for Research on Cancer (IARC) and the World Health Organization (WHO) as a comprehensive assessment of the global cancer burden. The latest iteration, conducted as GLOBOCAN 2020, estimated the incidence, mortality, and prevalence of 36 types of cancer stratified by sex and age groups in 185 countries and territories by 2020 (32). The results and methodology of the baseline study have been published in a previous study (1, 33). The GLOBOCAN study used the International Classification of Disease (ICD, 10th revision, version 2010) codes to estimate the epidemiology and burden of cancer according to their location. In this study, the results were reported using BC data (code: C50). The steps and methods for estimating cancer incidence and mortality according to the GLOBOCAN 2020 study (34) are detailed in two flowcharts in Figure 1. The HDI values for 2020 were extracted from the United Nations Development Programme (UNDP) data repository (35). Current healthcare expenditure (CHE) as a percentage of gross domestic product (GDP) indicators for 2019 as the available previous year were extracted from the Global Health Observatory data repository of WHO (36). This study was designed and the results were reported in accordance with the Guidelines for Accurate and Transparent Health Estimates Reporting (GATHER) statement (37) and the Strengthening the Reporting of Observational Studies in Epidemiology (STROBE) statement (38).

### Study variables

As primary epidemiologic measures, the incidence and mortality of female BC were extracted, reported, and used to calculate MIR. Regarding MIR as an indicator of healthcare quality, a low value was defined as better cancer care in terms of screening, treatment, and overall disease management because more cancer deaths per new case of BC could be avoided in locations with such values (11). CHE as a percentage of GDP, abbreviated as CHE/GDP (%) in this study, was another study variable for which national values were used, except for 15 countries/territories without corresponding estimates for 2019. The definition of the CHE/GDP ratio indicates the level of financial resources allocated to health relative to other uses, shows the importance of the health sector to the overall economy, and specifies the societal priority given to health, measured in financial terms (36). HDI is a composite indicator of socioeconomic status that includes life expectancy at birth, expected and mean years of schooling, and gross national income (GNI) per capita in purchasing power parity (in $) (35). HDI in the low, medium, high, and very high quartiles was used to descriptively report incidence and mortality measures, except for China and India based on the GLOBOCAN methodology. In addition, HDI point values for each country were used for correlation tests except for 10 countries/territories without HDI estimates. The other three stratifications for reporting incidence, mortality, and MIR values in this study were six WHO regions (Africa, Americas, Eastern Mediterranean, Europe, Southeast Asia, and Western Pacific), six continents based on the GLOBOCAN methodology (Africa, Asia, Europe, Latin America and the Caribbean, North America, and Oceania), and four income levels of the World Bank (WB; low, low middle, upper middle, and high).

### Statistical analysis

The incidence and mortality of BC were reported in the form of numbers (an estimated 95% uncertainty interval (UI)), crude rates, age-standardized incidence rates (ASIRs), and age-standardized mortality rates (ASMRs), all at rates per 100, 000 population. The cumulative risk of incidence and mortality of BC for all ages (in percentage) was reported assuming no competing causes of death. Age group-based number, crude rate, and cumulative risk of the incidence and mortality of BC were reported for age groups at 10 year intervals (0–9, 10–19, …, 60–69, 70+). The MIR values for BC were defined and calculated as the crude rates of mortality to crude rates of incidence ratio for each location (11). For countries/territories with available data, the associations between BC ASIR, ASMR, and estimated MIR values and concordance values for HDI and CHE/GDP were conducted using the bivariate correlation test, and Pearson's correlation coefficient was reported based on three ranges of absolute values: strong (>0.5), moderate (between 0.5 and 0.3), and weak (< 0.3). The level of two-tailed statistical significance for the recruited tests was set at 0.05. Data cleaning, analysis, and visualization were performed using IBM SPSS statistical software version 16.0 (SPSS, Inc., Chicago, IL) and the R statistical package for Windows version 4.1.2 (https://cran.r-project.org).

## Results

### BC incidence and mortality

**Figure 2:**
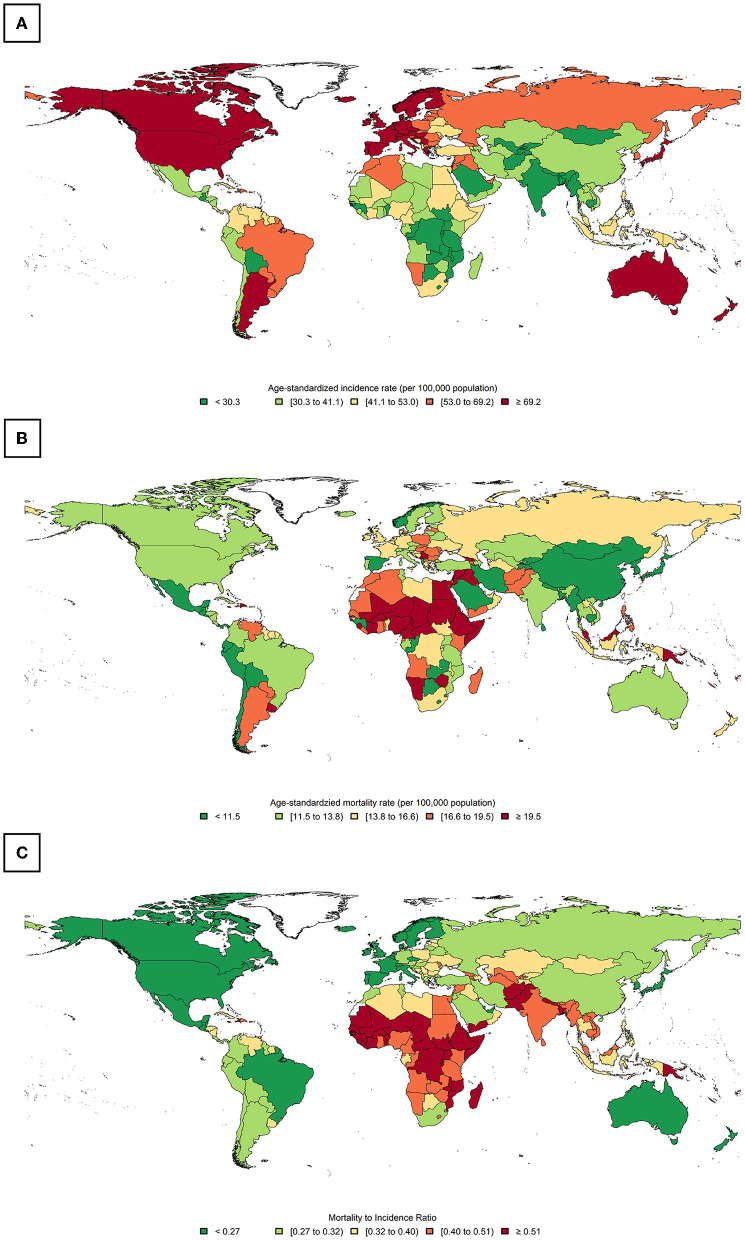
Global distribution of breast cancer (BC): **(A)** age-standardized incidence rate (ASIR), **(B)** age-standardized mortality rate (ASMR), and **(C)** the calculated mortality-to-incidence ratio (MIR).

Globally, there were 2,261,419 (95% UI: 2,244,260–2,278,710) new female BC cases in 2020, with a crude rate of 58.5/100,000 population and an all-age cumulative risk of 7.97%. BC led to 684,996 (675,493–694,633) deaths in 2020 with a crude rate of 17.7 and a cumulative risk of 3.14%. The WHO regions with the highest BC ASIR (69.7) were in Europe followed by Americas (68.0), and those with the lowest ASIR were in Southeast Asia (28.3), followed by Africa (38.7). The WHO regions with the highest BC ASMR were in Africa (19.1), followed by the Eastern Mediterranean (17.7), while those with the lowest BC ASMR were in the Western Pacific (10.5), followed by Southeast Asia (12.9). Continents with the highest and lowest BC ASIRs were in North America (89.4) and Asia (36.8), respectively. Furthermore, the ASMR for this categorization was the highest in Africa (19.4) and the lowest in Asia (11.9). WB income levels with the highest BC ASIR (81) were in the high-income quartile, while those with the lowest BC ASIR were in the low-middle income quartile (31), and the highest and lowest ASMRs were estimated for the low-income (18.3) and upper middle-income groups (12.1), respectively. For four-tier HDI categories (excluding China and India), the very high HDI category had the highest BC ASIR was the highest (75.6) in a very high HDI category against the lowest value in a medium HDI category (27.8), and ASMR was the highest in low HDI areas (20.1) vs. the lowest rate in the high HDI areas (12.1) (Table 1). BC ASIR among countries/territories ranked topmost in Belgium (113.2), The Netherlands (100.9), and Luxembourg (99.8) and bottom in Bhutan (5), The Republic of the Gambia (11), and Mongolia (11.1) (Figure 2A). Furthermore, BC ASMR was the highest in Barbados (42.2), Fiji (39), and Jamaica (34.1), while the rates were the lowest in Bhutan (2.6), Mongolia (3.9), and The Republic of the Gambia (5.8) (Figure 2B, Supplementary Table S1).

**Table 1:** Breast cancer (BC) incidence and mortality metrics in all-age number, crude rate, and age-standardized rates per 100,000 population, and an all-age cumulative risk for different geographic and socioeconomic categories.

**Location**	**Incidence**					**Mortality**					**MIR**
	**Number**	**Uncertainty interval**	**Crude rate**	**ASIR**	**Cumulative risk (%)**	**Number**	**Uncertainty interval**	**Crude rate**	**ASMR**	**Cumulative risk (%)**	
**World**	22,61,419	[2244260.0–2278710.0]	58.5	47.8	7.97	684,996	[675493.0–694633.0]	17.7	13.6	3.14	0.30
**WHO regions**											
**WHO Africa (AFRO)**	139,477	[128748.0–151100.0]	24.9	38.7	6.09	66,963	[59486.2–75379.5]	11.9	19.1	3.32	0.48
**WHO Americas (PAHO)**	491,691	[486206.0–497238.0]	94.8	68	11.61	106,391	[104052.0–108782.0]	20.5	13.2	3.23	0.22
**WHO East Mediterranean (EMRO)**	119,452	[113871.0–125306.0]	33.8	40.9	6.82	49,277	[46643.0–52059.7]	13.9	17.7	4.31	0.41
**WHO Europe (EURO)**	576,337	[561937.0–591106.0]	119.9	69.7	11.26	157,111	[150495.0–164018.0]	32.7	14.8	3.84	0.27
**WHO South-East Asia (SEARO)**	298,445	[289576.0–307586.0]	30.3	28.3	4.56	135,463	[129978.0–141179.0]	13.7	12.9	2.75	0.45
**WHO Western Pacific (WPRO)**	635,439	[625706.0–645324.0]	65.9	44.2	6.45	169,628	[164603.0–174806.0]	17.6	10.5	2.35	0.27
**Continent**											
**Africa**	186,598	[173041.0–201217.0]	27.8	40.7	6.53	85,787	[77648.4–94778.6]	12.8	19.4	3.91	0.46
**Asia**	10,26,171	[1019700.0–1032680.0]	45.3	36.8	5.61	346,009	[341537.0–350540.0]	15.3	11.9	2.57	0.34
**Europe**	531,086	[525074.0–537167.0]	137.2	74.3	11.86	141,765	[139102.0–144479.0]	36.6	14.8	3.9	0.27
**Latin America and the Caribbean**	210,100	[205031.0–215294.0]	63.2	51.9	8.69	57,984	[56734.5–59261.0]	17.4	13.5	3.15	0.28
**Northern America**	281,591	[280419.0–282768.0]	151.2	89.4	14.78	48,407	[47907.8–48911.5]	26	12.5	3.26	0.17
**Oceania**	25,873	[25370.0–26386.0]	121.4	87.8	14.2	5,044	[4801.1–5299.2]	23.7	14.7	3.7	0.20
**World Bank income levels**											
**Low income**	68,244	[63919.5–72861.1]	22.6	33.8	5.02	35,817	[31978.5–40116.2]	11.9	18.3	3.17	0.53
**Low middle income**	433,060	[417606.0–449086.0]	29	31	5.15	202,463	[192766.0–212647.0]	13.6	14.7	3.21	0.47
**Upper middle income**	880,235	[875935.0–884556.0]	60.6	44	6.58	259,216	[256857.0–261597.0]	17.8	12.1	2.68	0.29
**High income**	878,588	[872593.0–884624.0]	142.3	81	12.76	187,096	[184161.0–190077.0]	30.3	12.9	3.39	0.21
**HDI categories**											
**Low HDI**	109,572	[101016.0–118853.0]	22.2	36.1	5.67	58,586	[51225.0–67004.8]	11.8	20.1	3.62	0.53
**Medium HDI**	307,658	[297473.0–318191.0]	27.1	27.8	4.68	147,427	[140338.0–154874.0]	13	13.6	2.96	0.48
**High HDI**	825,438	[821342.0–829554.0]	57.2	42.7	6.35	247,486	[245212.0–249781.0]	17.2	12.1	2.68	0.30
**Very high HDI**	1,017,459	[1010050.0–1024920.0]	128.7	75.6	11.99	231,093	[227367.0–234880.0]	29.2	13.4	3.4	0.23

MIR, mortality-to-incidence ratio; WHO, World Health Organization; ASIR, age-standardized incidence rate; ASMR, age-standardized mortality rate; HDI, Human Development Index.

### MIR as an indicator of BC care quality

The global calculated female BC MIR in 2020 was 0.30. The ranking of MIR in WHO regions was the highest in Africa (0.48), followed by Southeast Asia (0.45), the Eastern Mediterranean (0.41), Europe (0.27), the Western Pacific (0.27), and the Americas (0.22). Among continents, Africa ranked top in MIR (0.46) and North America ranked bottom (0.17). Based on WB income levels, high- to low-order MIR of BC was low income (0.53), low-middle income (0.47), upper-middle income (0.29), and high income (0.21). High- to low-order MIRs in HDI quartiles (excluding China and India) were similar to those in low HDI (0.53), medium HDI (0.48), high HDI (0.30), and very high HDI (0.23) (Table 1). Countries with the highest MIR were Niger, Somalia, and the Central African Republic (all were 0.63). In contrast, countries with the lowest MRI were the Republic of Korea (0.12), Australia (0.16), and Finland (0.16) (Figure 2C, Supplementary Table S1).

### Findings in age groups

Globally, the 70+ age group had the highest BC incidence (194.8) and mortality (93) crude rates. Accordingly, the 70+ age group had the highest cumulative risk of BC incidence and mortality, with values of 3.85% and 2.02%, respectively. The latter age group had the highest MIR for BC (0.48), followed by the 60–69 group (0.31) and the 50–59 group (0.27). Excluding the first age group, the lowest MIR among age groups was in the 20–29 group (0.16), followed by the 30–39 group (0.17) and the 40–49 group (0.21) (Table 2).

**Table 2:** Global BC incidence and mortality metrics in all-age number, crude rate, cumulative risk, and MIR for age groups.

**Age group**	**Incidence**			**Mortality**			**MIR**
	**Number**	**Crude rate**	**Cumulative risk (%)**	**Number**	**Crude rate**	**Cumulative risk (%)**	
**0 to 9**	60	0.01	0	27	0	0	0.00
**10 to 19**	697	0.12	0	192	0.03	0	0.25
**20 to 29**	38,987	6.7	0.07	6,613	1.1	0.01	0.16
**30 to 39**	208,209	36.9	0.37	35,935	6.4	0.06	0.17
**40 to 49**	417,555	86.5	0.86	88,555	18.4	0.18	0.21
**50 to 59**	568,126	135.7	1.35	151,419	36.2	0.36	0.27
**60 to 69**	521,075	170.5	1.69	160,389	52.5	0.53	0.31
**70+**	506,710	194.8	3.85	241,866	93	2.02	0.48

MIR, mortality-to-incidence ratio.

### Associations between incidence, mortality, MIR, HDI, and CHE/GDP

**Figure 3:**
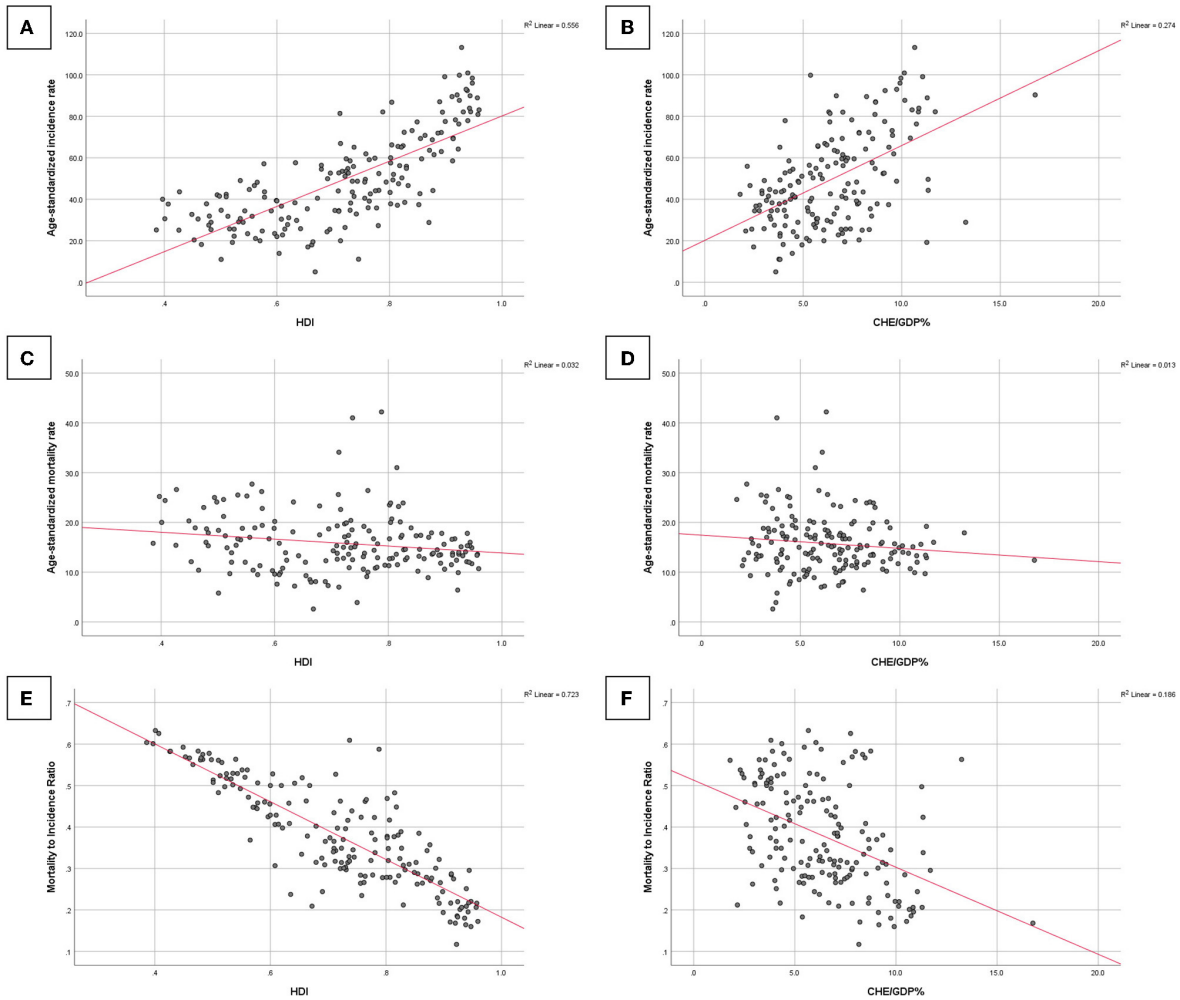
Associations of BC age-standardized incidence rate with **(A)** the human development index (HDI) and **(B)** the current healthcare expenditure to gross domestic product (CHE/GDP%), BC ASMR with **(C)** HDI and **(D)** CHE/GDP%, and BC MIR with **(E)** HDI and **(F)** CHE/GDP%.

A significantly strong correlation was observed between BC ASIR and the HDI and CHE/GDP values (Pearson's coefficients = 0.746 and 0.523, respectively, *p*-value < 0.001). A significant weak inverse correlation was observed between BC ASMR and HDI (Pearson's coefficient = −0.179, *p*-value = 0.018), while a weak and statistically non-significant correlation was observed between BC ASMR and CHE/GDP (Pearson's coefficient = −0.116, *p*-value = 0.132). A strong, statistically significant inverse correlation was observed between the MIR and HDI values for countries/territories with available data (Pearson's coefficient = −0.850, *p*-value < 0.001). Furthermore, a significant moderate inverse correlation was observed between the MIR and CHE/GDP values for locations with available data (Pearson's coefficient = −0.431, *p*-value < 0.001). Visualizations of the tested associations are shown in Figure 3.

## Discussion

This study attempted to investigate global BC epidemiology and care disparities globally, as well as the contribution of the level of development and healthcare expenditure to lower mortality rates compared to new cases of BC using MIR as an indicator of care. The main findings of this study suggested that BC ASIR was higher in more developed countries and those with higher income and healthcare expenditure, whereas mortality rates were higher in developing areas and those with limited income and healthcare expenditure. As an indicator of cancer care and management, the countries included in this study were ranked in terms of MIR. The MIR values were higher in countries and territories located in areas with low HDI or located in developing areas. The statistics were concerning for regions such as Africa, where the majority of the countries were among the top countries with the highest MIR values. Another important finding of this study was statistically significant associations between the MIR and HDI and CHE/GDP values calculated for countries and territories with available data, indicating that more developed areas and those that spend more on the healthcare systems, could improve BC outcomes and MIR as an indicator of cancer care.

One of the main findings of this study was consistent with similar epidemiologic literature studies in the field, suggesting that BC ASIR was higher in developed areas and countries of the world and in countries with higher income and healthcare expenditure. Previous studies on the Global Burden of Disease study and the sociodemographic index as a composite indicator of socioeconomic development found that BC incidence rates were higher in areas with higher development metrics (3, 4). This finding could be justified by population-wide BC screening programs that are more prevalent in developed countries, which will benefit from both well-designed health systems and higher expenditure in the health sector (40, 41). An ecological study from the USA deploying the age-period-cohort model to evaluate the contribution of mammography screening to trends in BC incidence reported that such screening plans led to a higher incidence of early stage invasive cancers in the population; however, widespread use of mammography also reduced the incidence of metastatic and advanced BC cases in the long term (39). A large-scale multinational epidemiologic investigation also approved the later finding on reductions in the incidence of advanced BC cases due to population-based mammographic screening (42). Although national wealth and development levels tend to be the primary reason for a higher BC incidence in such areas, one study showed that the incidence of BC is independently higher in these areas, highlighting the need for further research on BC incidence trends and influential factors (43).

BC ASMR was found to be high in developing regions, regions with low socioeconomic features, and limited resources in the general and health sections. These findings were consistent with previous studies, which suggested that the higher BC mortality rates in countries with rapid development and limited healthcare resources may predispose populations to barriers of access to and availability of BC early detection and timely treatment methods (3, 44, 45). In addition to the barriers to healthcare access, one study found that deficiencies in BC quality of care in the diagnosis and treatment stages are the main challenges in developing countries and in low- and middle-income countries suffering from the absence of cost-effective health policies, particularly cancer prevention and management (46). In this study, countries located in Africa had the highest rates of BC mortality, and the results are alarming. Evidence shows shortages of BC control programs in terms of early detection and limited access to updated therapeutic approaches in countries of this region, which are among the main challenges leading to such unfavorable outcomes for BC compared to other areas of the world (47–49). Considering existing disparities in BC care and outcomes, a reasonable approach to reducing the disparities in BC survival globally (50) could be the implementation of country-specific cost-effective interventions.

One notable result of this study was the use of MIR as an indicator of BC care, which could be successful in mapping disparities globally, as well as the notion that MIR was higher in areas with low development in HDI investigation. This finding is consistent with that of a study on BC epidemiology using MIR proxy from the Global Burden of Disease study data, which found that an inverse correlation was observed between the BC MIR and HDI values, with less developed countries having BC survival statistics (17). The concept of lower cancer MIR in more developed countries and regions has been examined for several malignancies like colorectal, gastric, prostate, oral cavity, kidney, and liver cancers, all of which indicated the impact of socioeconomic development on improving cancer outcomes from an epidemiological aspect (12, 13, 16, 19, 21, 24, 51). The findings of this study confirmed most of the studies that used MIR to study cancer care and evaluate healthcare disparities. A previous study on the use of MIR for pancreatic cancer reported no correlation between MIR variations and healthcare disparities among countries and suggested further research to evaluate this indicator (14).

It is necessary to have a more in-depth study of MIR as an indicator of quality of cancer care, as many other available proxies and indicators evaluated various healthcare systems and disease outcomes to reveal disparities and study key determinants. Recently, a developed measure of quality of care, similar to MIR, using MIR as an indicator in its composition is the Quality of Care Index (QCI) (10). The index has been implemented on epidemiological data for many causes of cancers, including BC and the responsible risk factors for better quality of care for BC in countries and regions with higher socioeconomic development added to improved management of BC risk factors, as the attributable burden was lower in more developed areas (2). The findings of such quality indicators are essential because cancer quality of care is closely related to disease outcomes and patient survival, and ensuring the quality of care in cancer is a priority for cancer management (52). Regarding BC, favorable care in more developed and wealthier countries mandates the implementation of best practices in developing countries and countries with limited resources *via* the provision of accessible and affordable healthcare services and increased BC awareness through health education and promotion programs (53, 54). In addition, it is essential to improve BC care by expanding the knowledge of research on quality of care of BC and evaluating the healthcare systems (55).

Findings on epidemiological measures of BC in association with HDI as a socioeconomic measure and CHE/GDP as a financial indicator of health systems were among the strengths of this study mapping existing disparities in BC care. As a composite indicator of life expectancy, education, and income of individuals, HDI is widely used in health studies and population-based investigations on BC epidemiology and outcomes stratified by this index to show the importance of social determinants of health in BC care (56, 57). One of these studies on BC and HDI suggested efforts to strengthen and optimize the performance of the healthcare system in less developed countries to address health disparities in BC care (58). From an economic perspective, the financial burden of cancer is enormous for healthcare systems and the affected patients. In developed countries, the economic burden of cancer is more studied, indicating a substantial burden on the healthcare systems that mandate appropriate policymaking and resource allocation to provide affordable cancer care (59–61). In addition, catastrophic healthcare expenditure and cancer out-of-pocket costs place a heavy burden on patients, which can substantially affect the quality of cancer care, especially in developing countries and low-income and low-resource countries (62–64).

The findings of this study have several implications for public health. Global, regional, and national efforts should focus on improving the quality of care provided to patients with BC, considering the growing trends of BC incidence and its significant burden. Empowering vulnerable regions and countries around the world with the worse findings of this study is needed to improve BC outcomes in these locations. Consideration of long-term socioeconomic improvements in less developed areas of the world *via* multisectoral policymaking and planning suggested a reduction in global disparities in BC care and management. Moreover, increasing the proportion of CHE/GDP through economic policies by governments and healthcare authorities is highly proposed to improve insurance and cost coverage for patients with cancer. In addition to the policies recommended to improve BC care, efforts to expand BC screening and prevention programs are needed to prevent the delayed presentation of patients in the advanced stage of the disease that does not warrant promising outcomes.

The main strengths of this study were the adoption of the most recent global cancer statistics on female BC, the mapping of MIR values as indicators of BC quality of care, and the presentation of disparities in BC care in different regions of the world, and the assessment of the associations of BC care with socioeconomic and financial factors. However, this study had some limitations. According to the GLOBOCAN methodology, the main limitation that needs to be declared is the quality and coverage of cancer data around the world, especially in low- and middle-income countries, which mandates careful interpretation of estimates (1, 33). The shortage of estimates for male BC was also limited by GLOBOCAN's data limitations. In addition, different BC histopathology data were not available to study BC burden based on tumor characteristics. Regarding HDI, the values for a limited number of countries/territories were not available, which could affect statistical analysis. In addition, the most recent CHE/GDP estimates were for 2019, and some countries lacked estimates.

## Conclusion

Disparities in BC care exist globally and vary by geography, socioeconomic development, and healthcare expenditure. Countries with low incomes, low development metrics, and limited financial resources for healthcare suffer more from the adverse outcomes of BC and have a less favorable quality of BC care. Countries and areas with a higher BC MIR need to improve the disease management course from diagnosis to treatment to alleviate the massive burden of female BC. In addition, enhancing the socioeconomic features of populations and empowering healthcare systems through increased allocation of resources would contribute to better BC care and augment the burden of this cancer on healthcare systems.

## Data availability statement

Publicly available datasets were analyzed in this study. This data can be found at: the GLOBOCAN study (available at: https://gco.iarc.fr/today). United Nations Development Programme (https://hdr.undp.org/data-center/human-development-index#/indicies/HDI). Global Health Observatory of World Health Organization [https://www.who.int/data/gho/data/indicators/indicator-details/GHO/current-health-expenditure-(che)-as-percentage-of-gross-domestic-product-(gdp)-(-)].

## Ethics statement

The studies involving human participants were reviewed and approved by the Research Ethics Committee of the Iran University of Medical Sciences (ID: IR.IUMS.REC.1400.1155). Written informed consent from the participants' legal guardian/next of kin was not required to participate in this study in accordance with the national legislation and the institutional requirements.

## Author contributions

SA: conceptualization, data curation, formal analysis, investigation, methodology, validation, visualization, project administration, writing—original draft, and writing—review and editing. SSM: data curation, formal analysis, investigation, visualization, and writing—review and editing. EM: methodology, validation, and writing—review and editing. SD and SR: resources and writing—review and editing. HRB: methodology, supervision, and writing—review and editing. MM: investigation, methodology, supervision, and writing—review and editing. All authors approved the final draft for publication.

## Supplementary material

Supplementary Table S1 National values of breast cancer (BC) incidence, mortality, mortality-to-incidence ratio (MIR), human development index (HDI), and current healthcare expenditure to gross domestic product ratio (CHE/GDP%) for 185 countries and territories included in the GLOBOCAN 2020 study.
